# N-Methylpyridinium Porphyrin Complexes as Sensitizers for Sonodynamic Therapy Against Planktonic and Biofilm-Forming Multidrug-Resistant Microbes

**DOI:** 10.3390/ijms26146949

**Published:** 2025-07-19

**Authors:** Daniel Ziental, Francesca Giuntini, Marcin Wysocki, Patrycja Talarska-Kulczyk, Agata Kubicka, Jolanta Dlugaszewska, Lukasz Sobotta

**Affiliations:** 1 Department of Inorganic and Analytical Chemistry, Poznan University of Medical Sciences, Rokietnicka 3, 60-806 Poznan, Poland; marcin.wysocki@student.ump.edu.pl; 2 School of Pharmacy and Biomolecular Sciences, Liverpool John Moores University, Liverpool L3 2AJ, UK; f.giuntini@ljmu.ac.uk; 3 Doctoral School, Poznan University of Medical Sciences, Bukowska 70, 60-812 Poznan, Poland; 4 Department of Immunobiology, Poznan University of Medical Sciences, Rokietnicka 8, 60-806 Poznan, Poland; patrycjatalarska@ump.edu.pl; 5 Department of Cancer Pathology, Greater Poland Cancer Centre, Garbary 15, 61-866 Poznan, Poland; akubicka@ump.edu.pl; 6 Department of Tumour Pathology and Prophylaxis, University of Medical Sciences, Garbary 15, 61-866 Poznan, Poland; 7 Department of Genetics and Pharmaceutical Microbiology, Poznan University of Medical Sciences, Rokietnicka 3, 60-806 Poznan, Poland; jdlugasz@ump.edu.pl

**Keywords:** porphyrins, SDT, PDT, antibiotic resistance, MRSA

## Abstract

Porphyrins play an extremely important role in both photodynamic (PDT) and sonodynamic therapy (SDT). These techniques, which have a lot in common, are largely based on the interaction between the sensitizer and light or ultrasounds (US), respectively, resulting in the formation of reactive oxygen species (ROS) that have the ability to destroy target cells. SDT requires the use of an appropriate frequency of US waves that are able to excite the chemical compound used. In this study, five porphyrin complexes were used: free-base meso-tetra(N-methyl-pyridinium-4-yl)porphyrin (TMPyP) and its transition metal complexes containing zinc(II), palladium(II), copper(II), and chloride-iron(II). The sonodynamic activity of these compounds was studied in vitro. The obtained results confirm the significant relationship between the chemical structure of the macrocycle and its stability and ability to generate ROS. The highest efficiency in ROS generation and high stability were demonstrated by non-metalated compound and its complex with zinc(II), while complex with copper(II), although less stable, were equally effective in terms of ROS production. Antibacterial activity tests showed the unique properties of the tested compounds, including a reduction in the number of both planktonic and biofilm antibiotic-resistant microorganisms above 5 log, which is rare among sonosensitizers.

## 1. Introduction

The potential of porphyrins is well known in the field of medicinal chemistry, particularly in terms of photodynamic and sonodynamic therapy (PDT and SDT, respectively), two similar techniques that rely on the interactions between the sensitizer and light or ultrasounds (US) to generate reactive oxygen species (ROS). For PDT, the mechanism is associated with direct absorption of photons by the sensitizer and its further excitation. The excited state is unstable due to excess energy and thus tends to return to the ground state via different pathways: emission of heat or light, or transformation to a more stable excited triplet state with lower energy. In this state, the sensitizer can react with surrounding molecules (Type I photodynamic process) or transfer its excess of energy to molecular oxygen (Type II photodynamic process). The drawback of such an approach is the requirement of light that usually has limited tissue penetration, being strongly dependent on the wavelength. In general, light at longer wavelengths provides better tissue penetration, although it can be insufficient for ROS generation [1,2,3,4,5]. In the case of SDT, the triggering processes are different. Excitation of the sensitizer occurs via the phenomenon of acoustic cavitation, oscillations of microbubbles in response to US at specific frequency and power. At high US intensities, the oscillations become irregular and the microbubbles tend to collapse, resulting in a high local temperature that promotes the formation of radicals from the affected molecules, such as the sensitizer, solvent etc. Radicals created in this way can then react with other surrounding molecules, but can also react with the sensitizer, resulting in its excitation. Additionally, SDT is associated with other effects, such as an increase in cellular permeability due to sonoporation [3,6,7,8]. Both methods (PDT and SDT) rely on specific chemical reactions that result in generation of particular ROS via two possible processes (Type I and II) [9]. In contrast to the sensitizer and natural oxygen, singlet oxygen is unstable and thus extremely reactive. Either radicals or singlet oxygen produced in Type I and Type II processes serve as potent oxidizers that are used for direct oxidation of the components of targeted cells, particularly the malicious ones, resulting in their destruction [3,9,10,11,12,13].

The mechanism of sonodynamic therapy (SDT) is not yet fully understood and it may never be completely elucidated. It appears to involve multiple chemical processes and physical phenomena that can occur simultaneously and complement each other. Besides the generation of ROS, partly induced by cavitation, recent studies emphasize the important roles of thermal and mechanical effects, which can cause damage to microbial cell membranes, enhance the uptake of sensitizers, or directly injure intracellular structures. The use of electron paramagnetic resonance (EPR) and selected fluorescence probes has confirmed the formation of hydroxyl radicals, superoxide anions, and hydrogen peroxide during sonication. The effectiveness of SDT largely depends on US parameters such as frequency, intensity, duty cycle, and pulse duration. Low-frequency US from 20 to 100 kHz promotes intense cavitation, whereas higher frequencies above 1 MHz favor thermal effects and more controlled release of ROS. The chemical structure of the sensitizer, including its lipophilicity, charge, and the presence of metal ions in the molecular core, also significantly influences the efficiency of the sonodynamic process. Studies on porphyrins and metalloporphyrins in SDT have shown several additional advantages. According to physical principles, low-intensity ultrasound cannot generate singlet oxygen in the same way as light does. However, it can cause cleavage of the branched chains of modified porphyrins, resulting in the production of other ROS such as hydroxyl radicals, hydrogen peroxide, and superoxide anions, all of which exhibit cytotoxic effects. Another advantage of porphyrins in PDT is their chemical modifiability, for example, through doping with metal ions, which reduces their light sensitivity. This allows for their use in SDT without the risk of phototoxicity and eliminates the need for patients to avoid sunlight after treatment. Porphyrins and their derivatives also hold strong potential as sonosensitizers in SDT and can be used as multimodal imaging agents, including magnetic resonance imaging, photoacoustic imaging, fluorescence imaging, and positron emission tomography. Importantly, many bacteria rely on porphyrins or their derivatives as essential metabolic cofactors that may facilitate the selective uptake of these compounds and thereby enhance the antimicrobial efficacy of SDT. In this paper, we focus on the potential of selected porphyrins as effective active substances against selected representative microorganisms characterized by multidrug resistance. The basic advantages of porphyrins as potential sonosensitizers include their wide availability and common natural occurrence. Porphyrins and their analogs occur in both the plant and animal world (e.g., heme, chlorophyll), which confirms their good safety profile and reduces the likelihood of acute toxicity. Additionally, as already mentioned, under the influence of US, porphyrins can generate, among others, hydroxyl radicals or superoxide anions, which have a high potential to destroy microorganisms without the risk of resistance development. It should also be emphasized that some porphyrins and their derivatives (e.g., HMME, PpIX, Ce6, DVDMS) have already been approved for clinical use in PDT, what facilitates their translation for applications in SDT [14]. From this perspective, it seems particularly interesting to better understand the relationship between the central metal of porphyrins and their antimicrobial effectiveness.

**Figure 1:**
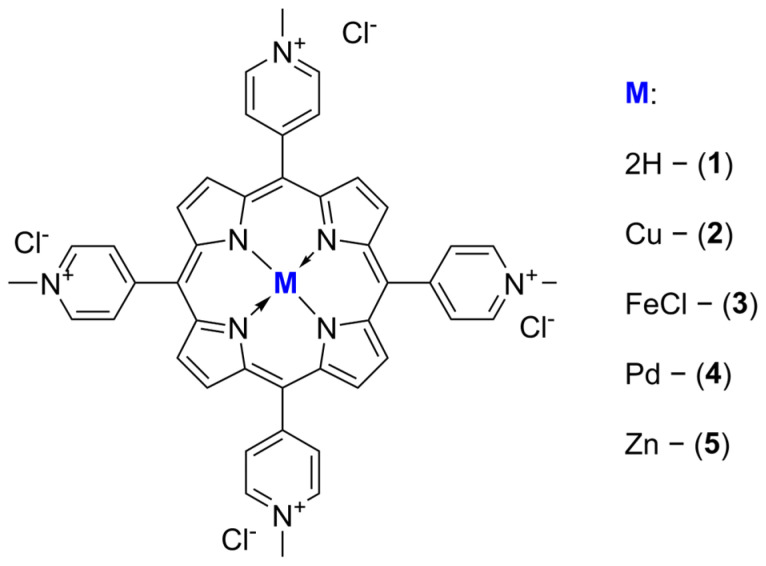
The structures of the porphyrins investigated.

The importance of these methods is associated with emerging difficulties in the treatment of bacterial infections. Since antibiotics have been systematically overused over the years, the resistance phenomenon of multi-drug resistant (MDR) bacteria had a chance to develop. This has resulted in a gradual decrease in the efficacy of antibiotics and increasing danger due to more difficulty in the treatment of resistant bacteria [3,6,15,16]. Notably, numerous studies have demonstrated the extremely low likelihood of microorganisms developing resistance to short-lived ROS [17]. In light of this, the development of therapeutic strategies based on PDT or SDT appears especially promising in the context of the looming crisis posed by pan-drug resistance. Herein, we present the 5,10,15,20-tetrakis(N-methylpyridinium-4-yl)porphyrin (TMPyP) along with its metallic complexes (Figure 1) and explore their sonodynamic activity toward bacteria in vitro.

## 2. Results and Discussion

The sonodecomposition process proceeded according to the first-order kinetic model, and for the assessment and comparisons the parameters were calculated using Equation (1).

ln[A0] = kt + ln[A](1)

**Figure 2:**
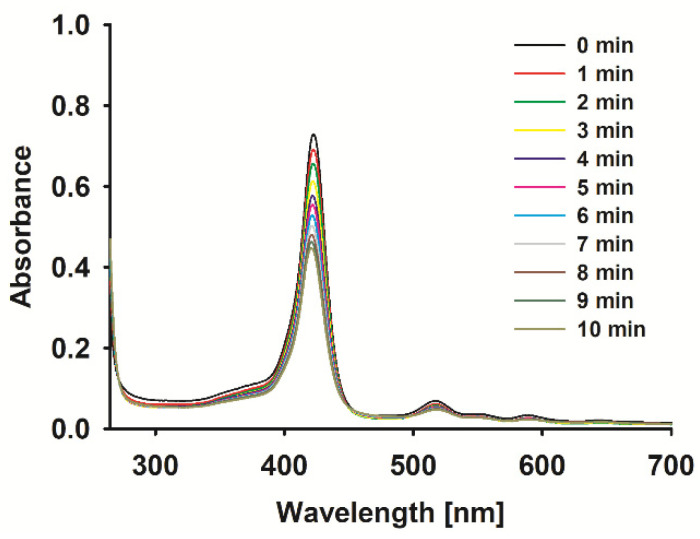
Spectra of 4 during sonication with ultrasound at 1 MHz and 3 W (40% duty cycle) in DMF.

The obtained sonostability kinetic parameters (Table 1) show that the incorporation of a metal ion meaningfully impacts the stability of the tested molecules. Ions Cu^2+^, FeCl^2+^ and Pd^2+^ significantly accelerate the macrocycle decomposition process from ca. 3-fold for porphyrin **2** up to over 13-fold for **4** (Table 1) in comparison to the non-metalated form (Table 1). Interestingly, the zinc(II) porphyrin (**5**) was more stable than its demetalated counterpart (**1**), what may be a result of the formation of, i.e., dimers by coordination bonds between zinc(II) central ions and peripheral substituents rich in macrocyclic π-electrons or nitrogen atoms. The dramatic drop in the stability of palladium(II) porphyrin (Figure 2) may be a result of the increased torsions in the molecular ion structure thanks to its large size, as exists above the macrocyclic ring. The sonostability of porphyrin **1**–**4** are comparable to those reported for zinc(II) phthalocyanine [18] and the model sonosensitizers of rose bengal and chlorin e6 [8].

**Figure 3:**
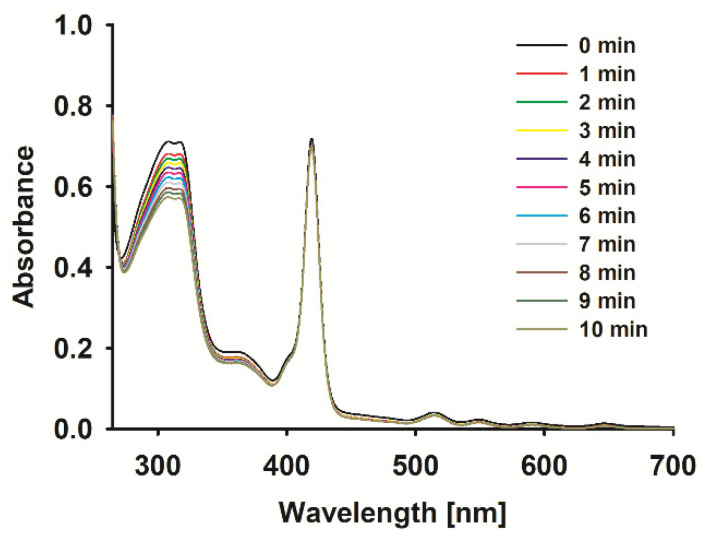
Spectra changes of **1** with TTF mixture during sonication (1 MHz, 3 W, 40% duty cycle).

**Figure 4:**
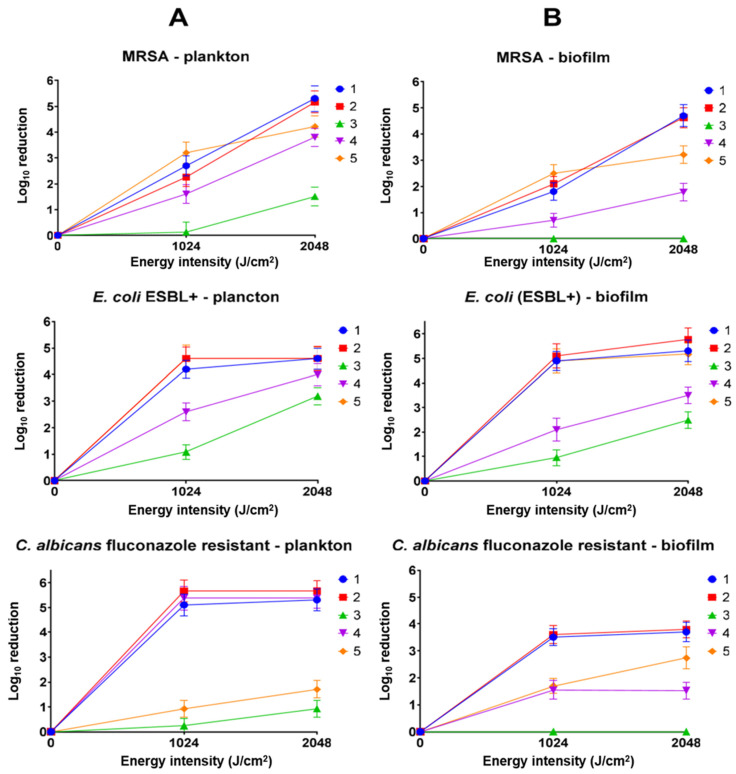
Antimicrobial activity of porphyrins **1**–**5** (1 MHz, 3 W, 40% duty cycle). (**A**) Activity against planktonic microorganisms; (**B**) activity against biofilm-forming microorganisms. The positive control with ultrasound showed no statistically significant difference from untreated microorganisms in the graph.

The ROS formation potential of the porphyrin complexes under study was evaluated with the aid of a chemical quencher. Due to the location of the studied porphyrins Soret bands, with a maximum light absorption in the range of 412–447 nm (Figure 3), and the absorption of the most common non-selective singlet oxygen chemical quencher—1,3-diphenylisobenzofuran (DBPF)—with a maximum absorption in the same range, tetratiafulvalene (TTF) with absorption at ca. 320 nm was chosen. In fact, the sonodynamic process is complex and many species are involved in the common interaction of many agents (sensitizer, solvent, quencher, etc.), resulting in a lot of ROS formation, heat effects, and many more [3]; as such, the kinetic parameters of quencher decomposition were evaluated (Table 2). The rate of TTF decomposition under US in the presence of a sensitizer increased for **1** (Figure 3), **4,** and **5** in comparison to the decomposition rate of the pure quencher. Interestingly, for **2,** the intensity of TTF degradation under US was comparable to the TTF sonostability, whereas compound **3** stabilized TTF. The compounds evaluated here have been previously tested with EPR spectroscopy for hydroxyl radicals and singlet oxygen generation under US influence. The ability to form both species with different yields was reported [19]. It is worth mentioning that the results obtained in the previous paper vs. those presented here are consistent. In the EPR measurements, activated with US zinc(II) porphyrin has been well recognized as the best ROS generator.

To the best of our knowledge, this is the first study to compare the effects of ultrasound-activated porphyrins on microorganisms in both planktonic and biofilm forms. Moreover, porphyrins with different central metal ions were utilized, enabling a comparison of the central ion’s influence on the efficacy of antimicrobial SDT [14,19]. Representative and model examples were used for evaluation, including Gram-positive bacteria (MRSA), Gram-negative bacteria (*E. coli* ESBL+), and fungi (fluconazole-resistant *C. albicans*). The research demonstrated that non-metalated and copper(II) porphyrins were particularly effective, reducing the number of each strain by at least 5 log (Figure 4). The concentration used, 0.5 mM, was the lowest concentration revealing activity above a 3 log reduction (the conventional limit for active antimicrobial compounds) [20,21]. This value qualifies them as powerful antimicrobial agents with significant potential for further development and study. In contrast, palladium(II) and zinc(II) porphyrins exhibited lower efficacy, showing a clear strain-dependent activity. Chloride–iron(II) porphyrin exhibited no antimicrobial activity. The lack of activity of chloride–iron(II) porphyrin correlates strongly with the results of sonochemical studies, where it acts protectively on a free radical quencher (Table 2). Additionally, unlike metal ions such as copper(II) or palladium(II), chloride–iron(II) ions did not enhance ROS production. Furthermore, under certain conditions, the released chloride-iron(II) ion may hypothetically promote microbial growth [22,23].

The application of SDT in combating microorganisms remains a relatively novel approach, with limited and inconsistent comparative data available [3]. For example, meso-tetra(carboxyphenyl) porphyrin (TCPP) was used against *S. aureus* in previous studies, achieving a 1.3 log reduction at a concentration approximately half of that used in the present study, also employing US at 1 MHz [24]. In another study, hematoporphyrin was used against *S. aureus*, resulting in about a 3log reduction (1 MHz, 6 W/cm^2^). However, this study underscores how early the development of SDT is, as the application of similar conditions (1 MHz, 6 W, and comparable exposure times [25]) in our experiment with a different device led to the eradication of MRSA without the use of a sonosensitizer.

Infections associated with the presence of microorganisms in biofilm form remain a significant challenge in the face of growing antibiotic resistance. It is currently estimated that over 80% of bacterial infections are linked to biofilm formation [26]. Biofilm formation is associated with an increased resistance of microorganisms to external factors, including antibiotics, disinfectants, and the host’s immune response. This resistance arises from the particularly dense, compact, and heterogeneous structure of pathogens, as well as their ability to produce extracellular polymeric substances that shield them from external agents [26]. Developing agents capable of effectively penetrating biofilms and inactivating microorganisms should be a priority in the search for new active substances with antibiotic or antibiotic-like properties. Previous studies indicate that results obtained in vitro against planktonic microorganisms do not always correlate with outcomes observed in biofilm formations, both in PDT and traditional antibiotic treatments [27]. However, the present findings do not exhibit such a disadvantage, particularly for the most active porphyrins: non-metalated, copper(II), and zinc(II) porphyrins (**1**, **2**, **3**, **4** and **5**, respectively). For both MRSA and *E. coli* (ESBL+), activity against planktonic and biofilm forms is comparable and high, reaching 5 log reduction or more. Greater differences were observed for fluconazole-resistant *C. albicans*. The activity of non-metalated, copper(II), and palladium(II) porphyrins decreased from values above a 5 log reduction to approximately a 3.5 log reduction, and to below a 2.7 log reduction for the remaining molecules. A characteristic feature of *C. albicans* biofilm formation is the presence of *α*-mannan, *β*-1,6-glucan, and *β*-1,3-glucan [28]. Although *β*-1,3-glucan occurs in the biofilm in relatively small amounts, it is considered a critical polysaccharide of the matrix, as it is associated with biofilm resistance to antifungal drugs by impeding drug diffusion. This is a distinct feature that differentiates fungal biofilms from bacterial ones, where the dominant component of the cell wall in most bacteria is peptidoglycan. Additionally, it is worth noting that *β*-glucans, unlike peptidoglycan, exhibit intrinsic antioxidant properties [29].

**Figure 5:**
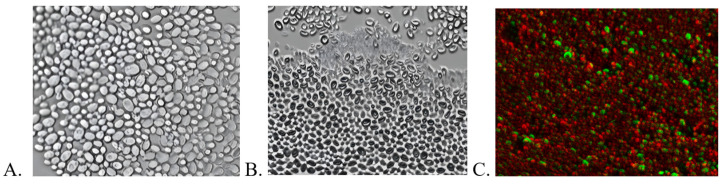
Imaging of fluconazole-resistant *C. albicans* biofilm using fluorescence microscopy. (**A**) Untreated biofilm. (**B**) Biofilm incubated for 30 min with copper porphyrin (partial staining of the cell observed). (**C**) *C. albicans* biofilm subjected to SDT with copper porphyrin (2048 J/cm^2^, 1 MHz, 3 W, 40% duty cycle); cells stained with Syto 9 and propidium iodide; visualization performed using the Apotom technique.

Despite more than 30 years of research on the effects of US on microorganisms, many aspects remain speculative. Based on available reports, it can be assumed that ultrasound effects may be thermal or non-thermal, although the boundary between these effects is difficult to clearly define [30]. In the presented study, the solution temperature in the measurement well did not exceed 40 °C at any point during the procedure. This indicates that the overall thermal effect should not significantly impact the viability of microbial cells. However, local rapid temperature increases resulting from sonochemical processes, which cannot be detected using traditional thermosensors, cannot be entirely ruled out [31]. Importantly, in the control samples, the application of ultrasound alone, without porphyrins, did not cause any harmful effects on the cell cultures. Combined with the results of TTF decomposition tests (Table 2), there is strong evidence to suggest that the bactericidal and fungicidal properties of the selected porphyrins are due to the generation of ROS during the sonodynamic process. This finding aligns with the previously mentioned higher resistance of biofilms formed by yeast, which are rich in potentially antioxidative *β*-glucan [29]. Fluorescence microscopy imaging indicates that ultrasound under the conditions applied in this study does not cause mechanical disruption of the biofilm (Figure 5), which explains the lack of antimicrobial effect in the absence of a sonosensitizer.

This phenomenon is typical for lower frequencies and higher energy levels. Therefore, the observed bactericidal effect in this case must result from the sonodynamic effect rather than mechanical cell damage caused by the sound wave [26].

**Figure 6:**
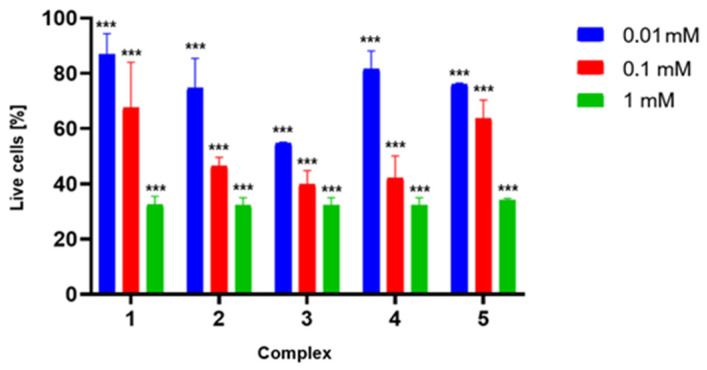
Results of the 7-AAD assay. Toxicity of porphyrins **1**–**5** against MRC-5 cells. The chart shows the percentage of viable cells after a 3 h incubation with porphyrins at various concentrations (0.01, 0.10, and 1.00 mM, respectively). Data are presented as a mean ± SD; *** for *p* > 0.001.

To confirm the potential for further development of the studied porphyrins, a preliminary toxicity study was conducted to evaluate their effects on fibroblasts (Figure 6). Each compound analyzed exhibited a relatively similar safety profile, causing a reduction in fibroblast cell viability by approximately 15–25% at a concentration of 0.01 mM. As the concentration increased, a correlated rise in toxicity was observed, culminating in approximately 70% reduction in cell viability at a concentration of 1 mM. Although this toxicity is relatively high, it should be noted that this study only correlates with real clinical scenarios to a limited extent. Firstly, in a potential clinical application, the porphyrin would be applied externally within the diseased area. Secondly, it should be remembered that the human body is highly adept at metabolizing porphyrins, heme, and their derivatives. Furthermore, the compounds did not exhibit toxicity toward the HT-29 (colorectal cancer) and HDF (human dermal fibroblast) cell lines tested by Giuntini et al. in a separate experiment, indicating that the sensitivity of different cell lines may vary significantly [19].

The authors anticipate that the developed compounds would be applied topically for such infections, with formulations designed specifically for local use. This makes it difficult to estimate the true concentration of the compounds in situ. Additionally, a short preincubation time limits penetration into mammalian tissues while maintaining activity against microorganisms. From this perspective, the significance of toxicity under ultrasound activation in eukaryotic cells is less relevant at this stage, as its assessment could lead to misleading conclusions about the toxicity or lack thereof. Furthermore, it should be noted that in the study by Giuntini et al., toxicity to normal cells under ultrasound exposure was significantly lower than to cancer cells, suggesting an exceptionally favorable safety profile [19].

## 3. Materials and Methods

Studied porphyrins **1** and **3**–**5** (Figure 1) were synthesized following previously reported procedures [19], and compound **2** was synthesized as reported by Pasternack et al. [32].

### 3.1. Sonochemistry Measurements

#### 3.1.1. TTF Decomposition Under Sonication in the Presence of the Sensitizer

The measurements were performed according to a method described previously [6,8,18]. Briefly, the decomposition of TTF in the presence of the studied porphyrins upon sonication was performed at ambient temperature in dark conditions in DMF solutions. The mixtures of sensitizers and TTF were aerated before experiment. The changes in mixture spectra were monitored with a Shimadzu U-1900 spectrophotometer (Shimadzu, Kyoto, Japan). The samples were sonicated in the ultrasonic apparatus constructed by the Institute of Fundamental Technological Research, Polish Academy of Sciences, Warsaw, Poland, equipped with an ultrasonic transductor (1 MHz, 3 W, 40% duty cycle).

#### 3.1.2. Sonostabilty of Sensitizers

The sonostability measurements were performed according to a method described previously [6,8,18]. Briefly, the sonostability of porphyrins was measured in DMF at ambient temperature under dark conditions. The solutions of the tested compounds were aerated before the experiments. Spectral changes were monitored with a Shimadzu U-1900 spectrophotometer. The samples were sonicated in the ultrasonic apparatus, equipped with an ultrasonic transductor (1 MHz, 3 W, 40% duty cycle).

### 3.2. Antimicrobial Assay

#### 3.2.1. Microorganisms and Growth Conditions

The microorganisms used in the study were clinical isolates of bacteria: methicillin-resistant *Staphylococcus aureus* (MRSA), *Escherichia coli* producing extended-spectrum beta-lactamases (ESBL+), and fungi: *Candida albicans* resistant to fluconazole. Bacterial and yeast strains were stored in Microbank cryogenic vials (ProLabDiagnostics, Richmond Hill, ON, Canada) at −70 ± 10 °C. The bacteria were cultured in brain heart infusion broth (BHI, OXOID, Cheshire, UK) at 36 ± 1 °C for 18 h. *C. albicans* cultures were grown in Sabouraud dextrose broth (SDB, Merck, Darmstadt, Germany) at 36 ± 1 °C for 24 h.

#### 3.2.2. Determination of the Dark Toxicity of Porphyrins on Planktonic Cells

The microorganisms were harvested by centrifugation and resuspended in a 0.9% solution of NaCl to a final concentration of about 10^7^ colony-forming units (CFUs)/mL. Aliquots (100 µL) of a standardized microbial suspension were placed in the wells of microtiter plates, and solutions of the sonosensitizers were added to give final concentrations of 0.5 mM. Negative control wells contained the microbial suspension in 0.9% NaCl without the porphyrins. All the samples were incubated in the dark for 30 min, and then the number of viable microbial cells in the samples was determined with the plate count method, using tryptic soy agar (TSA, OXOID, UK) for bacteria and Sabouraud agar (SA, OXOID, UK) for *C. albicans*. The log reduction in viable microorganisms was calculated based on the number of colony-forming units (CFUs) evaluated.

#### 3.2.3. Sonodynamic Inactivation of Planktonic Cells

The samples were prepared as described above. After incubation in the dark, the samples were subjected to US with a frequency of 1 MHz and a total energy of 1024 and 2048 J/cm^2^. Temperature was monitored during the experiment using a standard probe, with measurements recorded at 60 s intervals (Thermometer ST-80; Termoprodukt Poland, Bielawa, Poland). Viable microbial cells were measured similarly to dark toxicity to evaluate the antimicrobial activity. The number of CFUs was calculated, and the reduction in viable cells was determined in relation to the control samples containing microbes and saline alone.

#### 3.2.4. Determination of the Dark Toxicity of Porphyrins on Microbial Biofilm

Overnight cultures of bacteria and yeasts were diluted in a suitable medium to a final concentration of about 10^5^ CFU/mL. A total of 100 µL of diluted microbial suspensions were inoculated into 96-well flat-bottom microtiter plates and incubated at 36 ± 1 °C for 24 h. After incubation, the culture medium was discarded from the microtiter plate, and the biofilms formed on the plates were washed three times with saline to remove the planktonic cells. Subsequently, either 100 µL of sonosensitizers solution or 100 µL of saline were added, and the plates were incubated in darkness at room temperature for 30 min. Next, the biofilms were carefully scraped and homogenized. The number of surviving organisms and the log reduction in microbial cells for each sample were calculated using the plate count method, as described in the determination of the dark toxicity of porphyrins on planktonic cells.

#### 3.2.5. Sonodynamic Inactivation of Microbial Biofilm

The samples were prepared as described in the determination of the dark toxicity of porphyrins on microbial biofilm. After incubating the samples in the dark at room temperature, the samples were sonicated with a frequency of 1 MHz and a total energy of 1024 and 2048 J/cm^2^. Temperature was monitored during the experiment using a standard probe (Thermometer ST-80; Termoprodukt Poland), with measurements recorded at 60 s intervals. Next, the biofilms were scraped and homogenized, and the number of surviving microorganisms in the treated samples was determined using the plate count method to calculate the log reduction in microbial cells.

#### 3.2.6. Assessment of Biofilm Formation Using Fluorescence Microscopy

Biofilm formation was assessed using a ZEISS Axio Observer 7 (Zeiss, Jena, Germany) fluorescence microscope, equipped with Apotom for enhanced imaging. The visualization of biofilm formation was carried out using LIVE/DEAD™ BacLight™ Bacterial Viability Kits (Invitrogen, Carlsbad, CA, USA), following the manufacturer’s instructions.

### 3.3. Toxicity Evaluation

#### 3.3.1. Cell Line

In the studies, an established cell line of human fibroblasts MRC-5 was used. The cells were grown in Dulbecco’s modified Eagle’s medium (DMRM 1×, Corning, Corning, NY, USA) supplemented with 10% fetal bovine serum (FBS, Capricorn Scientific, Ebsdorfergrund, Germany)) and 1% of a mixture of the antibiotics penicillin G and streptomycin (Penicillin, Streptomycin Solution, 100×, Corning) on plastic plates (VWR Tissue Culture Plates, 24 wells, sterile). Cells were grown at 37 °C in an atmosphere of 5% CO_2_ with elevated humidity. After 24 h of cell culture, DMEM medium was replaced by the tested compounds, respectively (**1**–**5**), at three different concentrations (10^−3^, 10^−4^, 10^−5^) for the next 30 min, 1 h and 3 h. After that, cells were washed with phosphate-buffered saline (PBS, Dulbecco’s PBS (1×) w/o Ca and Mg, w/o Phenol Red, Capricorn scientific), detached from EDTA buffered with 0.25% trypsin solution (Trypsin-EDTA 0.25% in HBSS (1×) with Phenol Red, Capricorn Scientific), washed, and centrifuged. Cells with culture media without any tested compounds were the negative controls.

#### 3.3.2. Cells Viability

Staining with 7-Amino-actinomycin D (7 AAD), a viability dye which intercalates into double-stranded nucleic acids, was performed to evaluate the percentage of dead cells in the tested samples. Pre-collected, washed, and centrifuged cells were used to perform the viability test. Then, 5 µL of 7AAD was added to each tube containing 100 µL (10^5^) cell pellets resuspended in phosphate-buffered saline (PBS) and incubated for 10 min, protected from light and at room temperature. Each test trial was performed in duplicate. All samples were acquired with FACS Aria sorter (Becton Dickinson, Franklin Lakes, NJ, USA), and the results were analyzed with FACS Diva v6.1.2 Software (Becton Dickinson). An unstained sample was used as a control for the test. All experiments were conducted in six biological replicates.

## 4. Conclusions

The data presented in the article, in combination with previously conducted studies, confirm the potential of porphyrins as versatile compounds in sonodynamic therapy. Importantly, a significant effect of the central metal on the stability of the tested compounds and their ability to generate reactive oxygen species (ROS) is demonstrated. A clear correlation was observed between the type of central atom in the porphyrin derivatives and their ability to generate ROS under ultrasonic activation, with non-metalated and zinc(II)-substituted compounds consistently showing superior stability and sonodynamic efficiency compared to other metal-containing analogs. Copper(II) porphyrin proved to be clearly less stable but still very effective in terms of ROS production. The screening results of the antimicrobial activity of the tested compounds are exceptionally promising, showing very similar activity against both planktonic and biofilm microorganisms, achieving reductions exceeding 5 log for copper(II) porphyrin. This phenomenon is extremely desirable and rare among most sonosensitizers. What is particularly interesting and important is that the use of ultrasound alone did not lead to the physical destruction of the biofilm structure, as confirmed by fluorescence microscope images. This indicates a high degree of penetration by the tested compounds. These results, combined with excellent water solubility and a relatively good safety profile, suggest that the tested compounds are strong candidates for further studies in sonodynamic therapy for the treatment of superficial infections, both fungal and bacterial.

## Figures and Tables

**Table 1:** Kinetic parameters of sonosensitizer decomposition under ultrasounds exposition (1 MHz, 3 W, 40% duty cycle) in DMF.

Compounds	1	2	3	4	5
k (min^−1^)	0.04	0.01	0.012	0.049	0.003
t_0.5_	192.9	64.8	55.7	14.1	253.3
Ln (A)	0.021	0.02	0.012	0.011	0.021

**Table 2:** Kinetic parameters of TTF decomposition by sensitizer excited with ultrasounds [1 MHz, 3 W].

Compounds	TTF	TTF in the Presence of				
		1	2	3	4	5
k (min^−1^)	0.012	0.017	0.013	0.010	0.014	0.017
t_0.5_	59.4	41.3	52.0	70.574	48.301	41.360
Ln (A)	0.002	0.013	0.001	0.009	−0.004	0.009

## Data Availability

Dataset available on request from the authors.

## References

[1] Castro K.A.D.F., Ramos L., Mesquita M., Biazzotto J.C., Moura N.M.M., Mendes R.F., Almeida Paz F.A., Tomé A.C., Cavaleiro J.A.S., Simões M.M.Q. (2021). Comparison of the Photodynamic Action of Porphyrin, Chlorin, and Isobacteriochlorin Derivatives toward a Melanotic Cell Line. ACS Appl. Bio Mater..

[2] Li L., Qin F., Wang Y., Zhang Z. (2024). Singlet Oxygen Generation Proportion from Triplet State of Porphyrin in Water. Chem. Phys..

[3] Wysocki M., Czarczynska-Goslinska B., Ziental D., Michalak M., Güzel E., Sobotta L. (2022). Excited State and Reactive Oxygen Species against Cancer and Pathogens: A Review on Sonodynamic and Sono-Photodynamic Therapy. ChemMedChem.

[4] Gamelas S.R.D., Moura N.M.M., Habraken Y., Piette J., Neves M.G.P.M.S., Faustino M.A.F. (2021). Tetracationic Porphyrin Derivatives against Human Breast Cancer. J. Photochem. Photobiol. B Biol..

[5] Li M.Y., Mi L., Meerovich G., Soe T.W., Chen T., Than N.N., Yan Y.J., Chen Z.L. (2022). The Biological Activities of 5,15-Diaryl-10,20-Dihalogeno Porphyrins for Photodynamic Therapy. J Cancer Res Clin Oncol.

[6] Wysocki M., Ziental D., Jozkowiak M., Dlugaszewska J., Piotrowska-Kempisty H., Güzel E., Sobotta L. (2023). Porphyrazine/Phthalocyanine Hybrid Complexes—Antibacterial and Anticancer Photodynamic and Sonodynamic Activity. Synth. Met..

[7] Rajchel-Mieldzioć P., Tymkiewicz R., Sołek J., Secomski W., Litniewski J., Fita P. (2020). Reaction Kinetics of Sonochemical Oxidation of Potassium Hexacyanoferrate(II) in Aqueous Solutions. Ultrason. Sonochemistry.

[8] Ziental D., Wysocki M., Michalak M., Dlugaszewska J., Güzel E., Sobotta L. (2023). The Dual Synergy of Photodynamic and Sonodynamic Therapy in the Eradication of Methicillin-Resistant Staphylococcus Aureus. Appl. Sci..

[9] Ogilby P.R. (2010). Singlet Oxygen: There Is Indeed Something New under the Sun. Chem. Soc. Rev..

[10] Yang B., Chen Y., Shi J. (2019). Reactive Oxygen Species (ROS)-Based Nanomedicine. Chem. Rev..

[11] Akbar A., Khan S., Chatterjee T., Ghosh M. (2023). Unleashing the Power of Porphyrin Photosensitizers: Illuminating Breakthroughs in Photodynamic Therapy. J. Photochem. Photobiol. B Biol..

[12] Um W., Pramod Kumar E.K., Lee J., Kim C.H., You D.G., Park J.H. (2021). Recent Advances in Nanomaterial-Based Augmented Sonodynamic Therapy of Cancer. Chem. Commun..

[13] Canaparo R., Foglietta F., Barbero N., Serpe L. (2022). The Promising Interplay between Sonodynamic Therapy and Nanomedicine. Adv. Drug Deliv. Rev..

[14] Chen J., Zhou Q., Cao W. (2024). Multifunctional Porphyrin-Based Sonosensitizers for Sonodynamic Therapy. Adv. Funct. Mater..

[15] Le Guern F., Ouk T.-S., Yerzhan I., Nurlykyz Y., Arnoux P., Frochot C., Leroy-Lhez S., Sol V. (2021). Photophysical and Bactericidal Properties of Pyridinium and Imidazolium Porphyrins for Photodynamic Antimicrobial Chemotherapy. Molecules.

[16] Tovmasyan A., Batinic-Haberle I., Benov L. (2020). Antibacterial Activity of Synthetic Cationic Iron Porphyrins. Antioxidants.

[17] Huang Y.-Y., Sharma S.K., Dai T., Chung H., Yaroslavsky A., Garcia-Diaz M., Chang J., Chiang L.Y., Hamblin M.R. (2012). Can Nanotechnology Potentiate Photodynamic Therapy?. Nanotechnol. Rev..

[18] Wysocki M., Ziental D., Biyiklioglu Z., Jozkowiak M., Baş H., Dlugaszewska J., Piotrowska-Kempisty H., Güzel E., Sobotta L. (2025). Non-Peripheral Octasubstituted Zinc(II) Phthalocyanines Bearing Pyridinepropoxy Substituents—Antibacterial, Anticancer Photodynamic and Sonodynamic Activity. J. Inorg. Biochem..

[19] Giuntini F., Foglietta F., Marucco A.M., Troia A., Dezhkunov N.V., Pozzoli A., Durando G., Fenoglio I., Serpe L., Canaparo R. (2018). Insight into Ultrasound-Mediated Reactive Oxygen Species Generation by Various Metal-Porphyrin Complexes. Free Radic. Biol. Med..

[20] Ziental D., Czarczynska-Goslinska B., Wysocki M., Ptaszek M., Sobotta Ł. (2024). Advances and Perspectives in Use of Semisolid Formulations for Photodynamic Methods. Eur. J. Pharm. Biopharm..

[21] Domínguez A.B., Ziental D., Dlugaszewska J., Sobotta L., Torres T., Rodríguez-Morgade M.S. (2025). Multicationic Ruthenium Phthalocyanines as Photosensitizers for Photodynamic Inactivation of Multiresistant Microbes. Eur. J. Med. Chem..

[22] Banin E., Vasil M.L., Greenberg E.P. (2005). Iron and Pseudomonas Aeruginosa Biofilm Formation. Proc. Natl. Acad. Sci. USA.

[23] Almeida R.S., Wilson D., Hube B. (2009). Candida Albicans Iron Acquisition within the Host. FEMS Yeast Res..

[24] Sun D., Pang X., Cheng Y., Ming J., Xiang S., Zhang C., Lv P., Chu C., Chen X., Liu G. (2020). Ultrasound-Switchable Nanozyme Augments Sonodynamic Therapy against Multidrug-Resistant Bacterial Infection. ACS Nano.

[25] Zhuang D., Hou C., Bi L., Han J., Hao Y., Cao W., Zhou Q. (2014). Sonodynamic Effects of Hematoporphyrin Monomethyl Ether on Staphylococcus Aureus in Vitro. FEMS Microbiol. Lett..

[26] Erriu M., Blus C., Szmukler-Moncler S., Buogo S., Levi R., Barbato G., Madonnaripa D., Denotti G., Piras V., Orrù G. (2014). Microbial Biofilm Modulation by Ultrasound: Current Concepts and Controversies. Ultrason. Sonochemistry.

[27] Barra F., Roscetto E., Soriano A.A., Vollaro A., Postiglione I., Pierantoni G.M., Palumbo G., Catania M.R. (2015). Photodynamic and Antibiotic Therapy in Combination to Fight Biofilms and Resistant Surface Bacterial Infections. Int. J. Mol. Sci..

[28] Nett J., Lincoln L., Marchillo K., Massey R., Holoyda K., Hoff B., VanHandel M., Andes D. (2007). Putative Role of β-1,3 Glucans in Candida Albicans Biofilm Resistance. Antimicrob. Agents Chemother..

[29] Kofuji K., Aoki A., Tsubaki K., Konishi M., Isobe T., Murata Y. (2012). Antioxidant Activity of β-Glucan. Int. Sch. Res. Not..

[30] Xu P.-Y., Kumar Kankala R., Wang S.-B., Chen A.-Z. (2023). Sonodynamic Therapy-Based Nanoplatforms for Combating Bacterial Infections. Ultrason. Sonochemistry.

[31] Kinoshita M., Hynynen K. (2006). Mechanism of Porphyrin-Induced Sonodynamic Effect: Possible Role of Hyperthermia. Radiat. Res..

[32] Pasternack R.F., Gibbs E.J., Villafranca J.J. (1983). Interactions of Porphyrins with Nucleic Acids. Biochemistry.

